# Supplementary material for: The bimodally expressed microRNA miR‐142 gates exit from pluripotency
Source: Mol Syst Biol. 2015 Dec 21;11(12):850. doi: 10.15252/msb.20156525 (PMC4704488; doi:10.15252/msb.20156525)
Supplement: Supplementary file 1 — Appendix [file MSB-11-850-s001.pdf]

Appendix to *The bimodally expressed microRNA miR-142 gates exit from pluripotency*

Hanna L Sladitschek, Pierre A Neveu

Contents

**Appendix Figure S1**	**2**
**Appendix Figure S2**	**3**
**Appendix Figure S3**	**4**
**Appendix Figure S4**	**5**
**Appendix Figure S5**	**6**
**Appendix Figure S6**	**7**
**Appendix Figure S7**	**8**
**Appendix Figure S8**	**9**
**Appendix Figure S9**	**10**
**Appendix Figure S10**	**11**
**Appendix Figure S11**	**12**
**Appendix Figure S12**	**13**
**Appendix Figure S13**	**14**
**Supplementary Materials and Methods**	**16**
**Supplementary References**	**18**

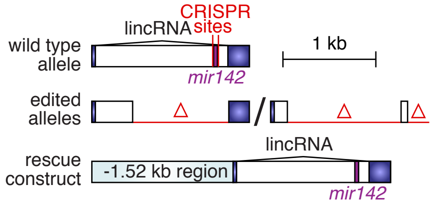

Appendix Figure S1. Generation of *mir142*$^{-/-}$ mESCs.

Generation of *mir142*$^{+/-}$, *mir142*$^{-/-}$ and *mir142*$^{-/-}$ rescue mESCs. Schemes of the alleles obtained using CRISPR/Cas9 technology and design of the rescue construct.

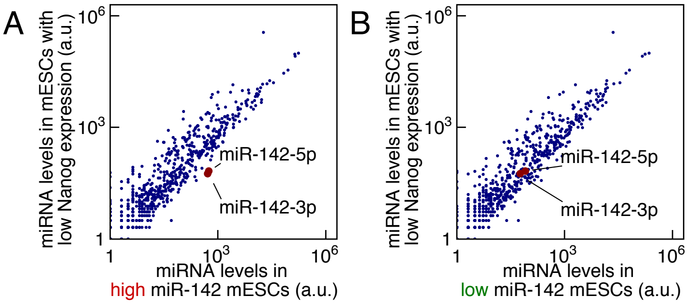

Appendix Figure S2. Comparison of miRNA expression levels in "high" and "low" miR-142 mESCs and mESCs with low Nanog expression.

A. Deep sequencing analysis of miRNA expression levels in FACS-purified "high" miR-142 mESCs and mESCs with low Nanog expression.

B. Deep sequencing analysis of miRNA expression levels in FACS-purified "low" miR-142 mESCs and mESCs with low Nanog expression.

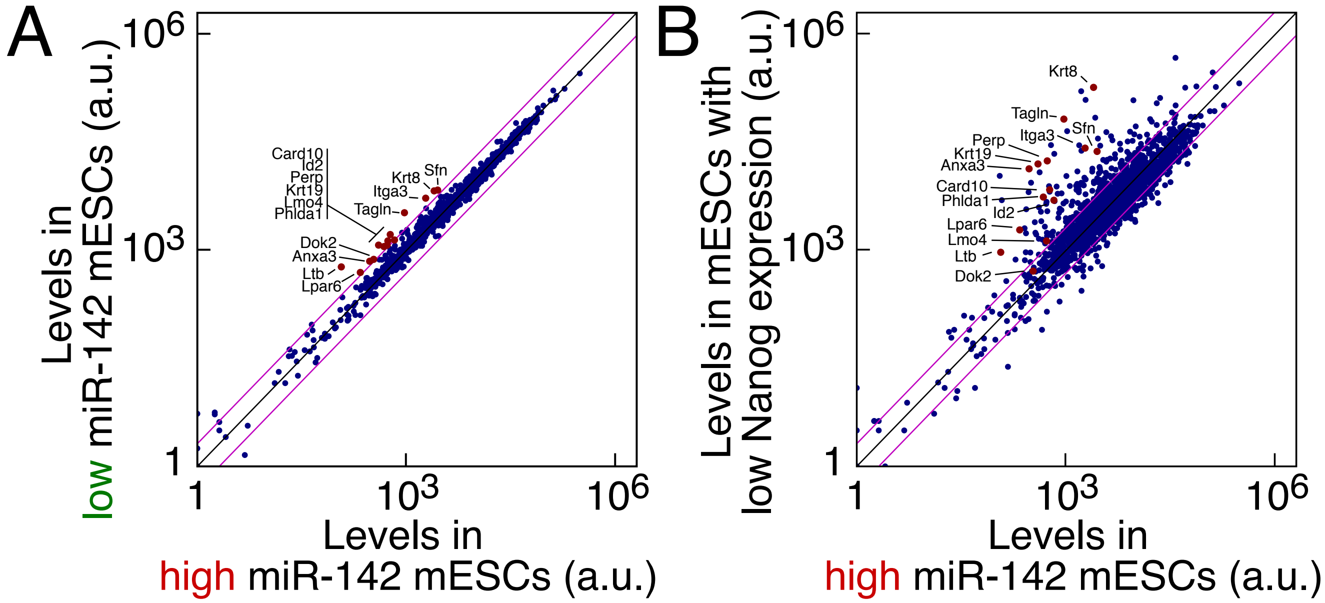

Appendix Figure S3. Expression levels of genes with reported variable expression in mESCs. .
A. mRNA expression levels of genes that have been identified as highly variable in mESCs by (Klein *et al*, 2015) in FACS-purified “high” and “low” miR-142 mESCs. 14 genes (highlighted in red) have more than 2-fold expression changes between “high” and “low” miR-142 mESCs.
B. mRNA expression levels of genes that have been identified as highly variable in mESCs by (Klein *et al*, 2015) in FACS-purified “high” miR-142 mESCs compared to mESCs with low Nanog expression. The 14 genes with more than 2-fold expression changes between “high” and “low” miR-142 mESCs are highlighted in red.

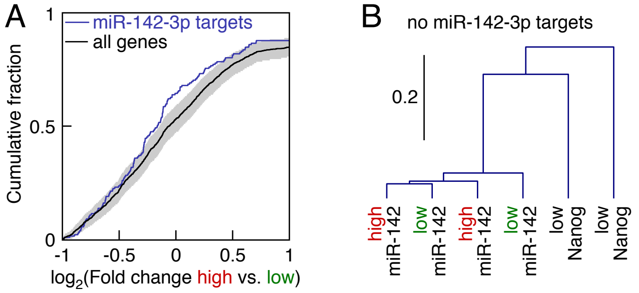

Appendix Figure S4. Expression levels of predicted targets of miR-142-3p in "high" and "low" miR-142 mESCs.

A. Cumulative distribution of gene expression changes in "high" miR-142 mESCs compared to "low" miR-142 mESCs (black line, gray area: 95% confidence interval). Blue line: distribution for the targets of miR-142-3p predicted by Targetscan (Grimson *et al*, 2007).

B. Average linkage hierarchical clustering of mRNA profiles of "high" and "low" miR-142 mESCs and of mESCs with low Nanog expression after removing miR-142-3p predicted targets.

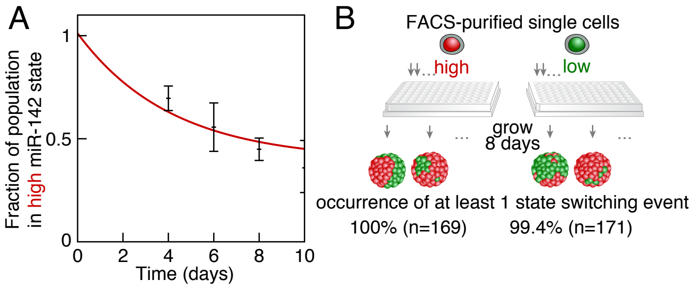

Appendix Figure S5. Interconversion between the two miR-142 states.

A. Fraction of cells in “high” miR-142 state during recovery of cultures started with FACS-purified “high” miR-142 state mESCs. Black: experimental data, error bars represent 95% confidence interval. Red line: one-parameter fit giving $k_1 + k_{-1} = 0.24 \pm 0.03$ per day.

B. Capacity of single mESCs or their progeny to switch state. Experimental scheme: Clonal cultures were derived from single FACS-purified “high” and “low” miR-142 mESCs. Occurrence of switching events was measured by assessing the miR-142 reporter ratio distribution in individual cultures.

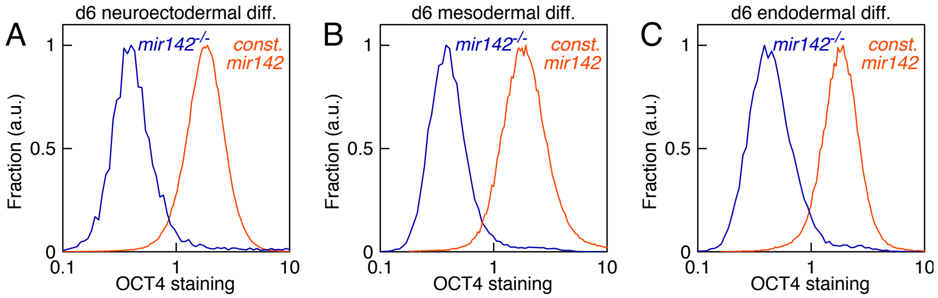

Appendix Figure S6. OCT4 expression in *mir142*$^{-/-}$ cells and cells with constitutive *mir142*-expression differentiated to neuroectoderm, mesoderm or endoderm fate.

A. Distribution of the protein expression levels of the pluripotency marker OCT4 in single *mir142*$^{-/-}$ cells (blue line) or cells with constitutive *mir142*-expression (orange line) differentiated for 6 days to neuroectoderm.

B. Distribution of the protein expression levels of the pluripotency marker OCT4 in single *mir142*$^{-/-}$ cells (blue line) or cells with constitutive *mir142*-expression (orange line) differentiated for 6 days to mesoderm.

C. Distribution of the protein expression levels of the pluripotency marker OCT4 in single *mir142*$^{-/-}$ cells (blue line) or cells with constitutive *mir142*-expression (orange line) differentiated for 6 days to endoderm.

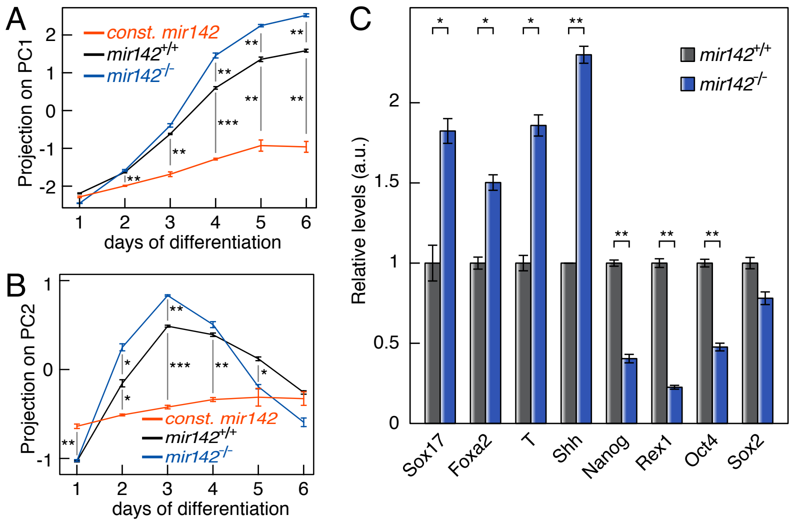

Appendix Figure S7. *mir142* expression locks mESCs in an undifferentiated state.

A-B. Projection on the first principal component PC1 (A) and the second principal component PC2 (B) of mRNA expression profiles during differentiation of wild type mESCs (*mir142*$^{+/+}$, black), *mir142*$^{-/-}$ mESCs (blue) or mESCs with constitutive *mir142*-expression (orange) (n= 2; *: $p < 0.05$, **: $p < 0.01$, ***: $p < 0.001$, two-sided t-test; error bars represent S.E.M.).

C. Endoderm and pluripotency marker expression in wild type cells (*mir142*$^{+/+}$, black) and *mir142*$^{-/-}$ (blue) cells differentiated for 6 days (n= 2, **: $p < 0.01$, ***: $p < 0.001$, t-test. Data represented as mean $\pm$ S.E.M.).

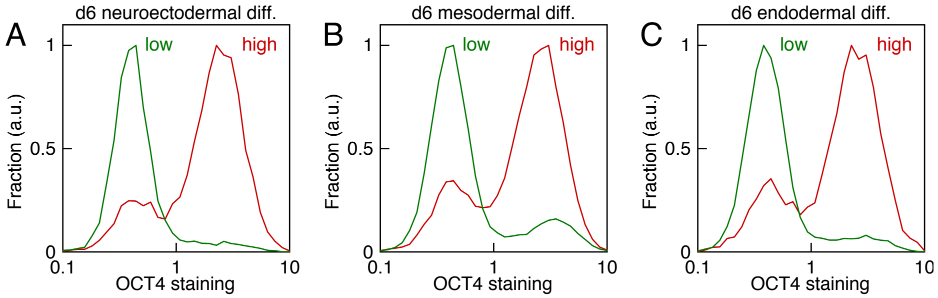

Appendix Figure S8. OCT4 expression in wild type mESCs differentiated to neuroectoderm, mesoderm or endoderm fate.

A. Distribution of the protein expression levels of the pluripotency marker OCT4 in single wild type cells differentiated for 6 days to neuroectoderm. Cells were classified as “high” (red line) or “low” miR-142 (green line) according to the miR-142 activity reporter signal.

B. Distribution of the protein expression levels of the pluripotency marker OCT4 in single wild type cells differentiated for 6 days to mesoderm. Cells were classified as “high” (red line) or “low” miR-142 (green line) according to the miR-142 activity reporter signal.

C. Distribution of the protein expression levels of the pluripotency marker OCT4 in single wild type cells differentiated for 6 days to endoderm. Cells were classified as “high” (red line) or “low” miR-142 (green line) according to the miR-142 activity reporter signal.

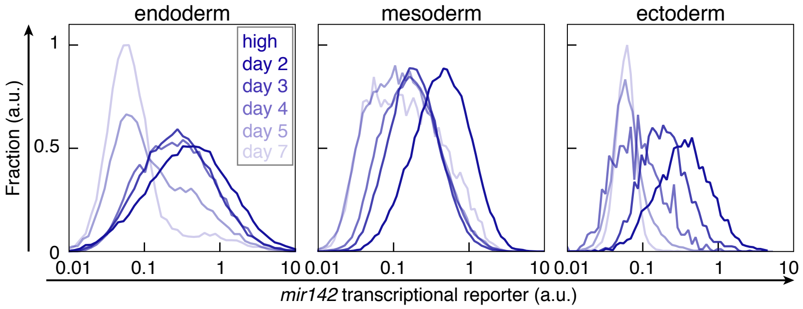

Appendix Figure S9. Evolution of *mir142* expression during differentiation.
Distribution of *mir142* transcriptional reporter expression in FACS-purified "high" miR-142 mESCs differentiated to endoderm, mesoderm and neuroectoderm. Shading denotes the time course of differentiation according to the boxed legend in the left panel.

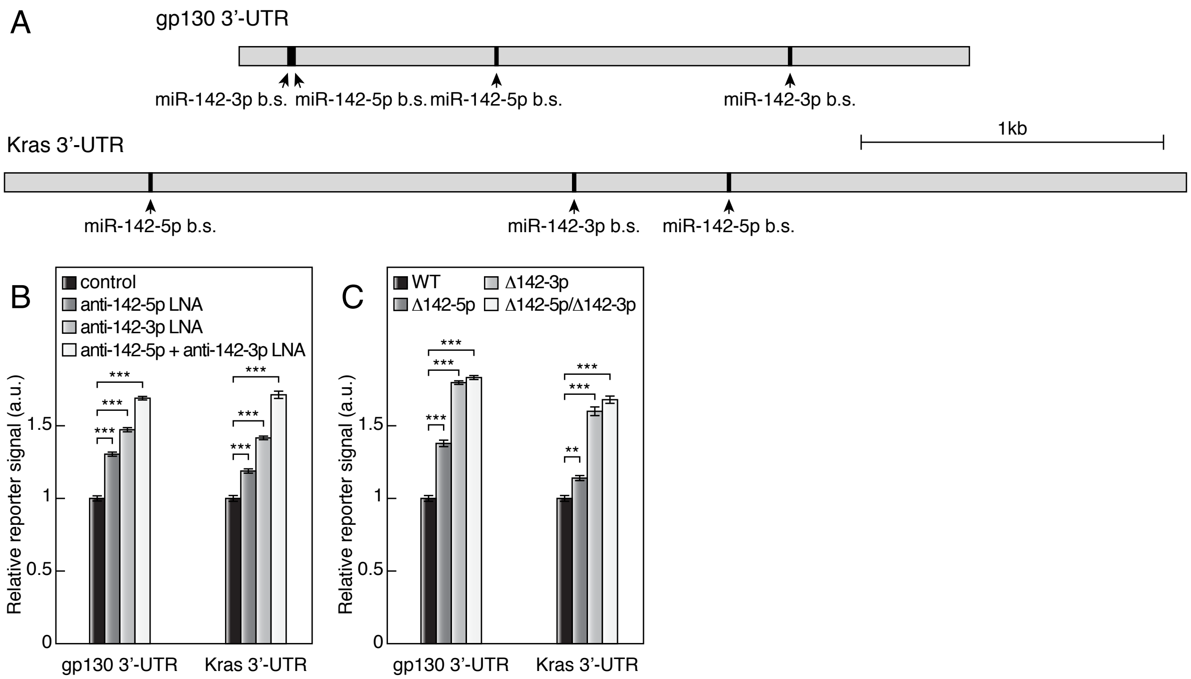

Appendix Figure S10. miR-142 targets gp130 and Kras in mESCs.
A. miR-142 target prediction. Targetscan predicts in the 3'-UTR of gp130 two binding sites for miR-142-3p and two binding sites for miR-142-5p (top panel). Targetscan predicts in the 3'-UTR of Kras one binding site for miR-142-3p and two binding sites for miR-142-5p (bottom panel). Bar: 1 kb; b.s.: binding site.
B. Reporter assay in mESCs using gp130 or Kras 3'-UTRs in the presence of LNAs against miR-142-3p or/and miR-142-5p or control LNA (n=6; ***: $p < 0.001$, two-sided t-test. Data represented as mean $\pm$ S.E.M.).
C. Reporter assay in mESCs using wild type gp130 or Kras 3'-UTRs (WT) or 3'-UTRs with deletion of all the seed regions of miR-142-5p (Δ142-5p), miR-142-3p (Δ142-3p) and both miR-142-5p and miR-142-3p (Δ142-5p/Δ142-3p) (n=6; **: $p < 0.01$, ***: $p < 0.001$, two-sided t-test. Data represented as mean $\pm$ S.E.M.).

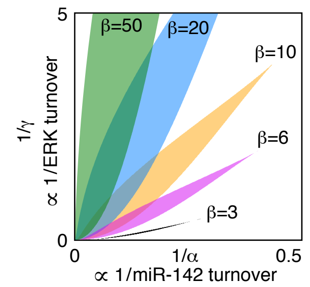

Appendix Figure S11. Theoretical phase diagram of the miR-142–ERK double-negative feedback loop for different values of β.

α represents a rescaled miR-142 turnover and γ an ERK activation turnover. The bistability region is colored differently for each value of β (black: β=3, magenta: β=6, yellow: β=10, blue: β=20, green: β=50).

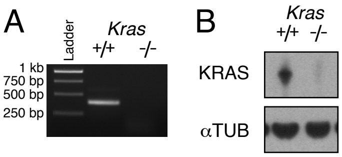

Appendix Figure S12. Generation of *Kras*$^{-/-}$ mESCs.

A. Genomic PCR on wild type mESCs (*Kras*$^{+/+}$) and *Kras*$^{-/-}$ mESCs with a primer specific for the first exon of *Kras*.

B. KRAS protein levels in wild type mESCs (*Kras*$^{+/+}$) and *Kras*$^{-/-}$ mESCs.

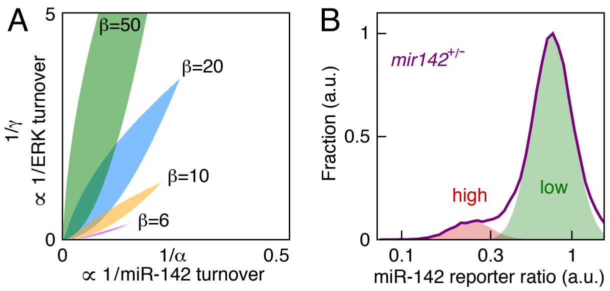

Appendix Figure S13. miR-142 activity in *mir142*$^{+/-}$ mESCs.

A. Parameter ranges that allow bistability in wild type and *mir142*$^{+/-}$ mESCs. The region is colored differently for each value of β (magenta: β=6, yellow: β=10, blue: β=20, green: β=50).

B. miR-142 reporter ratio in *mir142*$^{+/-}$ mESCs (purple line). *mir142* is bimodally expressed (“high” miR-142 state: red shaded area; “low” miR-142 state: green shaded area).

Supplementary Materials and Methods

Establishment of stable mESC lines

Plasmids were linearized and transfected using Fugene HD (Promega) according to the manufacturer's protocol. After antibiotic selection, single colonies were expanded and screened for expression of the transgene. All transgenic lines had normal karyotype.

Flow cytometry and fluorescence-activated cell sorting

Cells were dissociated to single-cell suspension with 0.05% Trypsin-EDTA (Invitrogen), resuspended in D-PBS, strained through a 40 μm cell strainer (BD Biosciences) and analyzed on an LSRFortessa flow cytometer (BD BioSciences). Subpopulations were purified or single cells were seeded into 96-well plates using a MoFlo sorter (DakoCytomation) or a BD Influx sorter (BD BioSciences). FACS-purified subpopulations were cultured for 16-20 h prior to sample preparation for immunoblot and for 24 h for alkaline phosphatase staining. Flow cytometry data was gated using the forward and side scatter signal to remove debris using FlowJo software and further analyzed using custom Python scripts. The logarithm of the miRNA reporter ratio was computed and adjusted by a sum of two gaussian distributions in order to quantify the number of cells in the “high” and “low” miR-142 states.

Analysis of proliferation rate

The CellTrace Violet cell proliferation kit (Invitrogen) was used according to the manufacturer's instructions and data was acquired on an LSRFortessa flow cytometer (BD BioSciences). miR-142 activity reporter was used to identify “high” and “low” miR-142 state cells. The distribution of the logarithm of CellTrace Violet signal was adjusted by a sum of gaussian distributions of fixed widths and fixed mean values corresponding to two-fold dilutions by cell division. Alternatively, “high” and “low” miR-142 mESCs were FACS-purified, plated in LIF+serum and cell numbers were counted every 8 hours for 3 days.

Alkaline phosphatase staining

The alkaline phosphatase detection kit (Millipore) was used following the manufacturer's protocol.

Western Blot

The following primary antibodies were used at the indicated dilutions under agitation overnight at 4°C: mouse monoclonal anti-AKT (pan) (Cell Signaling, 2920) at 1:1,000, rabbit monoclonal anti p-AKT (Ser473) (Cell Signaling, 4060) at 1:1,000, mouse monoclonal anti-ERK1/2 (Cell Signaling, 4696) at 1:2,000, rabbit monoclonal anti-p-ERK1/2 (Thr202/Tyr204) (Cell Signaling, 4370) at 1:2,000, rabbit monoclonal anti-GAPDH (Cell Signaling, 5174) at 1:400,000, rabbit polyclonal anti-gp130 (Santa Cruz, sc-656) at 1:3,000, mouse monoclonal anti-KRAS (Santa Cruz, sc-30) at 1:200, rabbit polyclonal anti-NANOG (Bethyl Lab, A300-397A) at 1:30,000, mouse monoclonal anti-OCT-3/4 (Santa Cruz, sc-5279) at 1:200, rabbit monoclonal anti-RAS (Cell Signaling, 8955) at 1:1,000, rabbit polyclonal anti-REX1 (Thermo Scientific, PA5-27567) at 1:1,000, mouse monoclonal anti-SOX2 (Cell Signaling, 4900) at 1:1,000, mouse monoclonal anti-STAT3 (Cell Signaling, 9139) at 1:1,000, rabbit monoclonal anti-p-STAT3 (Tyr705) (Cell Signaling, 9145) at 1:2,000, rabbit monoclonal anti-α-TUBULIN (Cell Signaling, 2125) at 1:5,000. The following secondary antibodies were used at the indicated dilutions under agitation for 1 h at RT: goat anti-mouse IgG (H+L) conjugated to horseradish peroxidase (Jackson ImmunoResearch, 115-035-146) at 1:50,000-1:200,000, goat anti-rabbit IgG (H+L) conjugated to horseradish peroxidase (Jackson ImmunoResearch, 111-035-144) at 1:80,000-1:200,000. Blocking and incubation with antibodies were carried out in 50 mg/ml BSA in TNT-buffer (100 mM Tris-Cl pH 7.5, 150 mM NaCl, 0.1% (v/v) Tween-20).

Immunostaining

Single-cell derived mESC colonies cultured on 0.1% gelatin-coated glass-bottom dishes (Lab-Tek, Nunc) or μ-slides (Ibidi) (for analysis by imaging) or mESCs dissociated to single-cell suspension with citric saline (135 mM KCl, 15 mM sodium citrate) (for flow cytometry analysis) were fixed in 4% PFA in D-PBS (15 min, RT) and permeabilized in TSB-buffer (1% (v/v) Triton-X 100, 2 mg/ml SDS, 10 mg/ml BSA in D-PBS) (1 h, RT). The following primary antibodies were used at the indicated dilutions under gentle agitation overnight at 4°C: anti-NANOG at 1:2,000, anti-REX1 at 1:1,000, anti-SSEA-1 (Santa Cruz, sc-21702) at 1:500 and anti-OCT-3/4 at 1:100, anti-DESMIN (Cell Signaling, 5332) at 1:100, anti-FOXA2 (Cell Signaling, 8186) at 1:400, anti-TUJ1 (Cell Signaling, 5568) at 1:200. The following secondary antibodies were used at the indicated dilutions and incubated for 2 h at RT: goat anti-mouse IgG (H+L), F(ab')2 fragment labeled with Pacific Blue dye (Invitrogen, P31581) at 1:500-1:1,000, goat anti-rabbit IgG (H+L) F(ab')2 fragment labeled with Alexa Fluor 647 (Cell Signaling, 4414) at 1:1,000-1:2,000. All antibodies were diluted in 50 mg/ml

BSA in TNT-buffer, which was also used as blocking agent prior to application of primary and secondary antibodies. Cells incubated with isotype controls were used as negative control. Data was acquired on an inverted SP8 confocal microscope (Leica) or an LSRFortessa flow cytometer (BD BioSciences).

Validation of miR-142 targets

3'-UTRs of mouse gp130 (Il6st) and Kras were cloned downstream of PGK::H2B-Citrine. All target sites for miR-142-3p and miR-142-5p were deleted using primers designed using the Quikchange Primer Design Program (Agilent). Transient transfections in mESCs were performed using Lipofectamine 2000 (Invitrogen) according to manufacturer's instructions. Control A, anti-miR-142-3p and anti-miR-142-5p LNA (Exiqon) were transfected at a total final concentration of 50 nM. A *PGK::H2B-Cherry-bGHpA* plasmid was used as transfection control at 1:4 ratio. Fluorescence signal was analyzed 24 h after transfection on an LSRFortessa flow cytometer (BD BioSciences).

Image segmentation and tracking

Images were first median filtered with a 3x3 pixel neighborhood and then gaussian filtered. Cells with diminishing intensities were segmented by increasing the standard deviation of the gaussian filter. The segmented image was built from the zero crossings of the Laplacian of the filtered image. Segmentation of the background was rejected based on pixel noise, average pixel intensity, blob size and shape. For cell tracking, images belonging to consecutive time frames were locally registered in order to obtain a local displacement field to guide cell assignment and correct segmentation if needed. Cells were matched between two time frames using a cost matrix based on distance, fluorescence intensity and blob size using the hungarian algorithm. Final tracking results were manually curated.

Supplemental References

Grimson A, Farh KKH, Johnston WK, Garrett-Engele P, Lim LP, Bartel DP (2007) MicroRNA targeting specificity in mammals: determinants beyond seed pairing. *Mol Cell* **27**: 91–105

Klein AM, Mazutis L, Akartuna I, Tallapragada N, Veres A, Li V, Peshkin L, Weitz DA, Kirschner MW (2015) Droplet Barcoding for Single-Cell Transcriptomics Applied to Embryonic Stem Cells. *Cell* **161**: 1187 – 1201
