# Supplementary material for: The bimodally expressed microRNA miR‐142 gates exit from pluripotency
Source: Mol Syst Biol. 2015 Dec 21;11(12):850. doi: 10.15252/msb.20156525 (PMC4704488; doi:10.15252/msb.20156525)
Supplement: Supplementary file 2 — Expanded View Figures PDF [file MSB-11-850-s002.pdf]

# Expanded View Figures

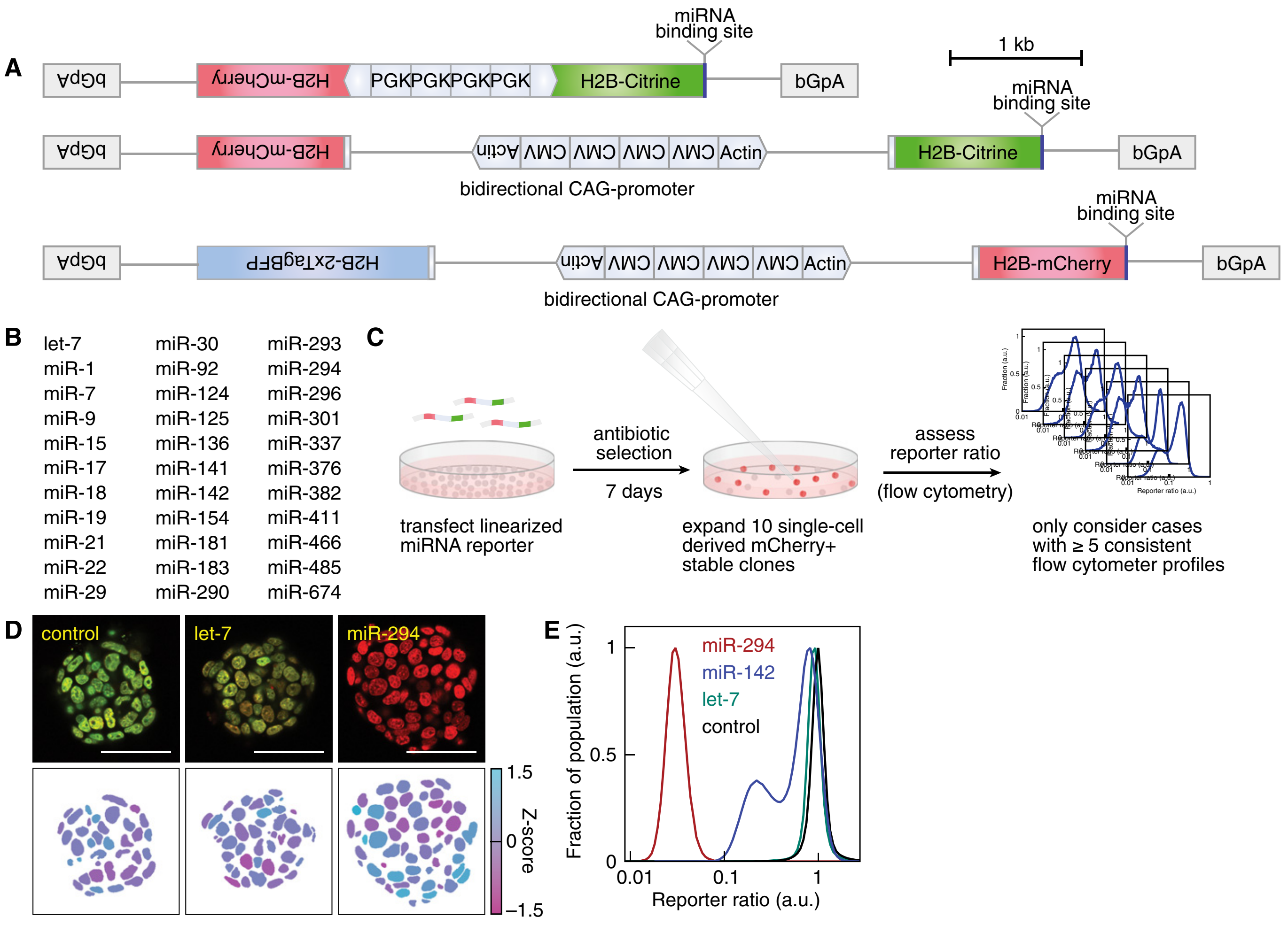


**Figure EV1. Single-cell miRNA activity reporter.**

A A synthetic bidirectional promoter drove the expression of H2B-Cherry as normalizer to control for transcriptional noise and H2B-Citrine as detector of miRNA activity with a target sequence for the respective miRNA 11 bp downstream of its stop codon. Four enhancer elements of the mouse phosphoglycerate kinase *Pgk1* gene (PGK) promoter were inserted between two back-to-back arranged minimal PGK-promoter fragments to create a bidirectional PGK promoter (upper panel). The bidirectional CAG-promoter was constructed by placing four CMV immediate-early enhancer elements between two back-to-back arranged fragments of the promoter, first exon and partial intron of chicken *β-actin* gene fused to the splice acceptor of the rabbit *β-globin* gene (lower panel). A positive selection cassette was included (not depicted). Intronic sequences are represented as lines. bGpA: rabbit *β-globin* genomic fragment containing the polyadenylation signal. For use in the *Rex1*-dGFP knockin mESC line, we constructed an activity reporter based on a bidirectional CAG-promoter driving the expression of H2B-2xTagBFP as normalizer and H2B-Cherry as detector.

B List of candidate miRNAs used in this screen.

C Experimental scheme for the generation and screening of clonal mESC lines stably expressing miRNA activity reporters.

D Reporter signal in single cell-derived mESC colonies and corresponding Z-score of the reporter ratio for a non-targeted control, a let-7a-5p and a miR-294-3p activity reporter. Scale bar: 50 μm.

E Reporter ratio distribution in mESCs stably expressing the activity reporters for miR-294-3p (red line), miR-142-3p (blue line), let-7a-5p (green line) and a non-targeted control (black line).

© 2015 The Authors

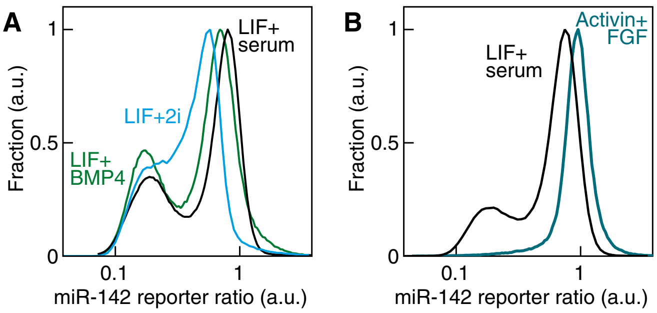


**Figure EV2. miR-142 activity reporter in different pluripotency-sustaining media.**

A Bimodal miR-142 expression in naïve LIF-dependent pluripotency conditions. Distribution of the miR-142-3p reporter ratio in mESCs cultured in 10 ng/ml LIF supplemented with serum (LIF+serum, black line) or 10 ng/ml BMP4 (LIF+BMP4, green line) or in 1 μM PD0325901 + 3 μM CHIR99021 (LIF+2i, blue line).

B miR-142-3p reporter ratio distribution in primed pluripotency conditions (12 ng/ml FGF2 and 20 ng/ml Activin A, blue line) compared to naïve LIF-dependent pluripotency conditions (LIF+serum, black line).

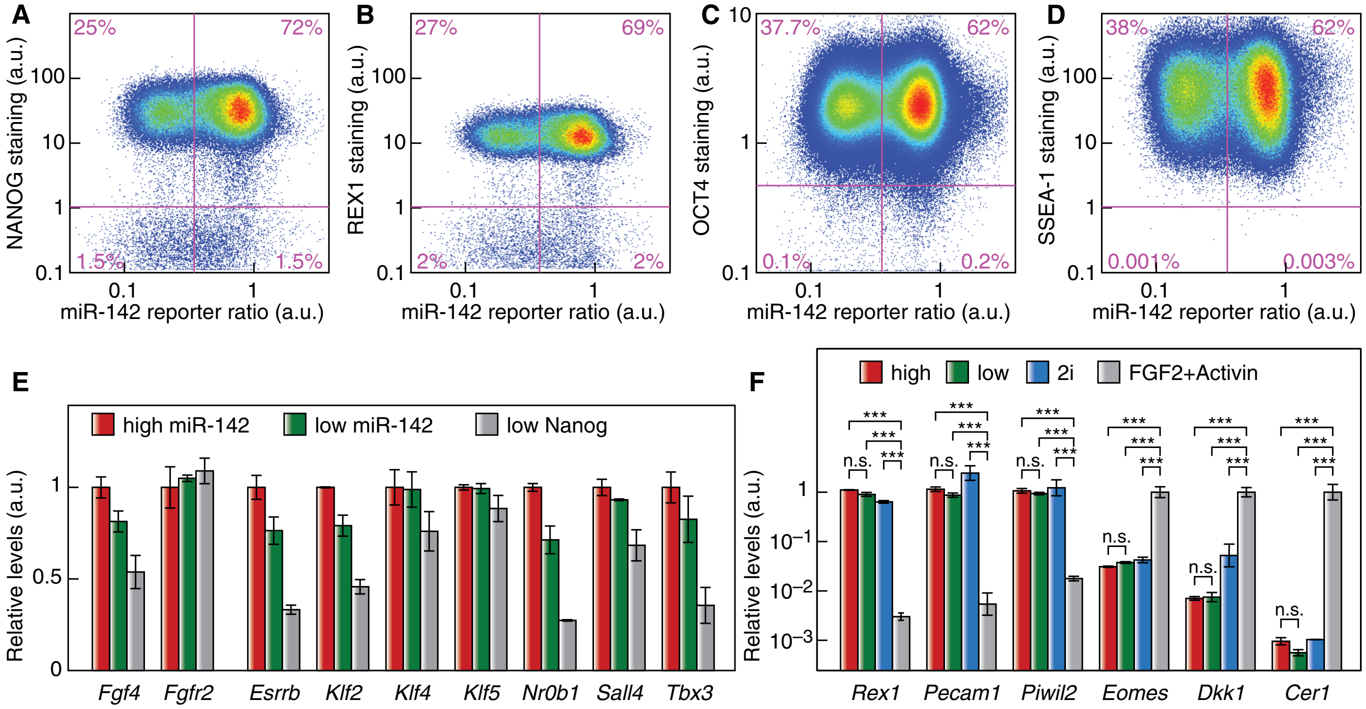


**Figure EV3. "High" and "low" miR-142 mESCs express pluripotency markers at equal levels and do not express epiblast stem cell markers.**

A–C Protein expression levels of the pluripotency markers NANOG (A), REX1 (B) and OCT4 (C) in single mESCs expressing the miR-142 activity reporter.

D Expression levels of the pluripotency marker SSEA-1 in single mESCs expressing the miR-142 activity reporter.

E mRNA expression levels of additional pluripotency markers in FACS-purified "high" and "low" miR-142 state mESCs as well as cells with low Nanog expression ($n = 2$; data represented as mean ± SEM).

F mRNA expression levels of mESC and epiblast stem cell markers in "high" and "low" miR-142 state mESCs as well as mESCs maintained in "2i" and epiblast stem cells maintained in primed pluripotency conditions (FGF2 + Activin) ($n = 2$; n.s.: not significant, ***$P < 0.001$, two-sided $t$-test). Data represented as mean ± SEM.


© 2015 The Authors

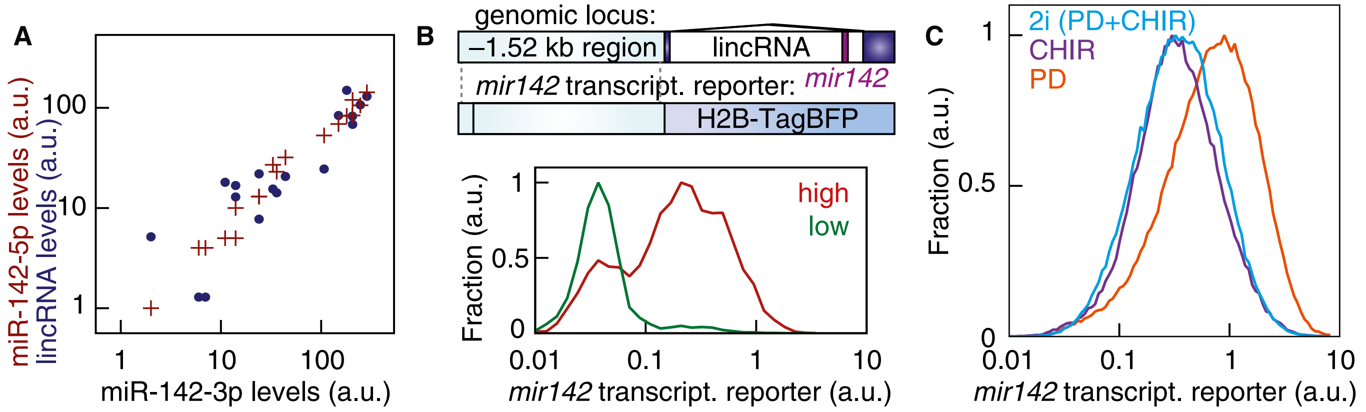


**Figure EV4.** ***mir142*** **transcriptional reporter.**

A Comparison between miR-142-3p, miR-142-5p and *mir142*-hosting lincRNA expression levels measured by deep sequencing across 18 matched mRNA-miRNA libraries. Levels of miR-142-3p were well-correlated with the levels of miR-142-5p and of the *mir142*-hosting lincRNA ($r$ = 0.989, $P$ = $10^{-14}$; $r$ = 0.881, $P$ = $10^{-6}$, respectively).

B Design of the fluorescent *mir142* transcriptional reporter and *mir142* transcriptional reporter signal in "high" and "low" miR-142 mESCs in double transgenic mESC lines expressing both the miR-142 activity and the *mir142* transcriptional reporters. The *mir142* transcriptional reporter was not expressed beyond background autofluorescence levels in the majority of "low" miR-142 mESCs but was expressed in the majority of "high" miR-142 mESCs confirming transcription as the source of the bimodal regulation of *mir142* expression.

C Distribution of *mir142* transcriptional reporter expression in the presence of 1 µM PD0325901 (PD, orange line) or 3 µM CHIR99021 (CHIR, purple line) or the combination of both inhibitors ("2i", blue line). GSK-3 inhibition counteracted the effects of ERK-inhibition on the *mir142* transcriptional reporter expression.

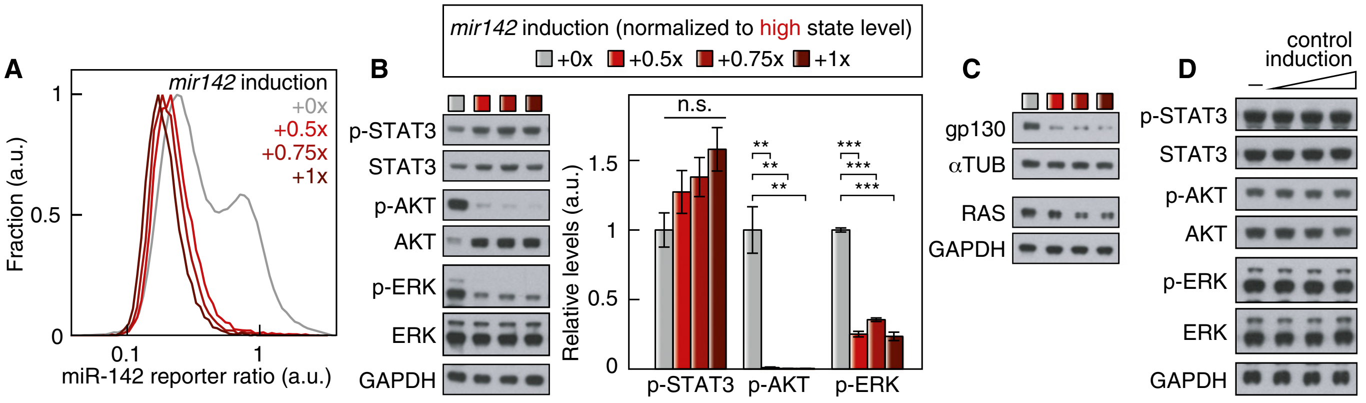


**Figure EV5. Effect of miR-142 gain-of-function on the activation of the STAT3, AKT and ERK signaling pathways.**

A Distribution of miR-142 reporter ratio at the three *mir142* induction levels used to assay the pathway activation status.

B Activation status of ERK, AKT, STAT3 upon *mir142* induction in mESCs and quantification ($n$ = 3; **$P$ < 0.01, ***$P$ < 0.001, two-sided $t$-test; error bars represent SEM). Induction levels are color-coded according to the boxed legend.

C gp130 and RAS protein levels upon *mir142* induction in mESCs. *mir142* induction levels are color-coded according to the boxed legend in (B).

D Activation status of ERK, AKT and STAT3 and their total levels upon induction of a control construct in mESCs.

© 2015 The Authors
