# Supplementary material for: The bimodally expressed microRNA miR‐142 gates exit from pluripotency
Source: Mol Syst Biol. 2015 Dec 21;11(12):850. doi: 10.15252/msb.20156525 (PMC4704488; doi:10.15252/msb.20156525)
Supplement: Supplementary file 3 — Movie EV1 [file MSB-11-850-s003.zip › Movie EV1 legend.rtf]

Movie EV1: Live imaging of state switching.Maximal projections of confocal sections during growth of a single-cell derived colony. Corresponds toFigure 3F and G. Time format: hh:mm. Bar: 50 μ. Time interval used during imaging = 15 to 30 min toaccommodate for colony growth in Z.
